# Is it worth publishing Open Access? – the scientific impact of Open Access publications in the field of medical education

**DOI:** 10.1080/10872981.2026.2652722

**Published:** 2026-03-30

**Authors:** Hendrik Friederichs

**Affiliations:** a Medical School OWL, Bielefeld University, Bielefeld, Germany

**Keywords:** Medical education, Open Access, bibliometrics, research article, scientific impact

## Abstract

**Introduction:**

In medical education research, the transfer of knowledge depends heavily on the visibility of scientific publications. Whether Open Access (OA) actually increases this visibility through a citation advantage (Open Access Citation Advantage, OACA) is still unclear for the field of medical education. This study aims to determine the existence and size of an OACA for medical education articles indexed in PubMed between 2010 and 2019.

**Method:**

In a retrospective bibliometric cohort study, all entries classified as research articles by iCite® with the MeSH term ‘Medical Education’ (*N* = 43,275) were analysed. OA was defined by the presence of a PubMed Central identifier (PMCID). Primary endpoints were total citations, citations per year, and the Relative Citation Ratio (RCR). Group comparisons were made using Yuen‑Welch t‑tests (20 % trimmed means, *α* = 0.05). Sensitivity analyses included negative binomial regression with year and journal fixed effects, and quantile regression for RCR.

**Results:**

21.2% of articles had a PMCID. PMCID-indexed publications showed higher trimmed means for total citations (10.31 vs. 7.00), annual citations (1.29 vs. 0.79), and RCR (0.79 vs. 0.49; all *p* < .001). Robust effect sizes (${\hat{\delta }}_{R}^{\text{AKP}}$, Algina-Keselman-Penfield robust standardized difference) ranged from 0.27 to 0.35 (small to medium). Negative binomial models with year and journal fixed effects confirmed these findings (IRR range: 1.53–1.67).

**Conclusions:**

PMCID-indexed articles in the field of medical education are cited significantly more frequently and have higher field‑normalised impact values than non‑OA publications. Despite financial hurdles and methodological limitations including potential selection bias, the moderate OACA supports strategically expanding OA publication funds to maximise the reach and impact of medical education research. Prospective studies should consider different OA types, altmetrics, and potential confounders to pinpoint the impacts of OA.

## Introduction

As scientists, our primary task is to create knowledge. It is not only of interest to acquire new knowledge, but also to ensure that this new knowledge is disseminated, i.e. read by other scientists and possibly also implemented in their work. This applies in particular to the field of medical education because new knowledge is applied in a variety of ways. The implementation of new knowledge not only improves the knowledge of the students, but also the teaching process itself. This raises the question of how the hard-won knowledge can be made available to other scientists as effectively as possible. There are two established forms of academic publication: One is the traditional form of publication, usually free of charge for the authors, via journals that sell the content to readers. On the other hand, there is the costly form of publication via journals that offer all or part of the content to readers for free while the authors need to pay the journals, known as open access. With both forms, there are obstacles that potentially make the transfer of knowledge more difficult, therefore, the scientists may want to carefully consider the form of publication they choose. This raises the central question: is it worth publishing Open Access?

Many lecturers and students need reliable evidence at all times in order to keep teaching and learning processes up to date. Nevertheless, traditional paywalls block a considerable part of the medical literature. This inadequate supply of information is also reflected in the education sector: teaching staff often only gain access to relevant studies years after they were published, which perpetuates outdated curricular concepts. Open Access (OA) models address the access problem, but shift the cost burden onto authors. Frank et al. explained that article processing charges (APCs) of several thousand US dollars structurally disadvantage young researchers and female academics from resource-poor regions in particular [1]. This asymmetry is also confirmed in global cross-sectional studies. Abdel-Razig et al. showed that the median APC in health professions education journals is USD 2,820 (as of 2024) and is up to ten times higher in relative terms for authors from countries with low purchasing power [2]. Forero et al. also warn of the danger of predatory journals discrediting the OA movement and spreading misinformation [3]. These developments highlight a power imbalance, as OA benefits readers but, APC-based models exacerbate social selectivity on the producer side [4]. Overall, evidence-based teaching is thus caught in a dilemma between knowledge equity and publication economics. This study addresses this area of tension for the field of medical education and examines whether OA generates measurable scientific added value despite financial hurdles.

Open access is not a monolithic concept but encompasses several distinct pathways. Gold OA refers to articles published in fully open-access journals, where authors typically pay article processing charges and content is immediately freely available under an open license. Hybrid OA describes individual articles made openly accessible within otherwise subscription-based journals, usually for an additional fee. Green OA involves author self-archiving of manuscripts in institutional or subject repositories, such as PubMed Central (PMC), often following a publisher-imposed embargo period. Finally, bronze OA denotes articles that are freely readable on publisher websites but lack formal open licensing, limiting reuse rights. These distinctions matter for bibliometric research because each pathway may differentially affect discoverability, readership, and ultimately citation patterns.

The question of Open Access Citation Advantage (OACA) has occupied bibliometric researchers for two decades. Langham-Putrow et al. analysed 134 primary studies and found that 47.8% reported a significant OACA, while 27.6% showed no effect and 23.9% demonstrated only selective benefits [5]. Hua et al. found citation advantage range of OA in biomedical fields from −5% to 83% citation difference, which indicates considerable heterogeneity [6]. Piwowar et al. supplemented this evidence in an analysis with large-scale samples of a corpus of 67 million publications. After adjusting for discipline and age, OA articles achieved 18% more citations [7]. Clark et al. also confirmed a mean advantage of 17.8 citations per OA publication in 146,415 biology articles [8]. The level of APC did not correlate with the citation frequency, which Yuen et al. already suspected for surgical journals, but were able to show a low correlation (r ≈ 0.25) for this area [9]. In medicine, cross-sectional analyses show a mixed picture. Björk and Solomon found no general difference in the citation rates between OA and subscription journals after controlling for journal age, discipline and country of publication [10]. At the article level, Yi et al. showed an OACA of an average of 25.08 additional citations of OA articles compared to subscription articles in the *Postgraduate Medical Journal* [11], whereas Davis et al. found no effect in a randomised design (minus 4% citation probability) [12]. Methodological factors explain some of these inconsistencies, as Basson et al. demonstrated that the choice of database shifted the measured OA proportion by up to 18% [13], and Atayero et al. showed that CiteScore distributions vary between OA and subscription sources in a subject-specific manner [14]. Overall, the literature suggests that OACA may exist, but that its extent is field- and time-dependent. To date, there has been no large-scale study of medical education—a gap this study attempts to fill.

Despite the subject dynamics in healthcare and higher education, it is unclear whether OA publications in medical education are actually cited more frequently than non-OA articles. Peidu et al. even found the opposite trend in the life sciences, where paid articles received 69% more citations [15]. At the same time, Logullo et al. point out that while OA increases transparency, it does not necessarily improve methodological quality [16]. Medical education research thus faces an evidence gap, as it is unknown whether OA in this specialised field offers a citation advantage that justifies the APC incurred and promotes didactic dissemination. To evaluate whether knowledge dissemination truly accelerates, raw citation counts alone are insufficient. This study therefore highlights the Relative Citation Ratio (RCR), a metric developed by the NIH that is field- and time-normalised at the article level, enabling equitable comparisons across specialties and publication years.

This study aims to determine the existence and size of an OACA for medical education publications. Specifically, for all research articles listed in PubMed between 2010 and 2019, this study examines whether the presence of a PubMed Central ID (PMCID)—as a surrogate for free full-text OA—is associated with

(1) higher absolute citation counts,
(2) more citations per publication year, and
(3) an increased relative citation ratio (RCR).

This study is differentiated from prior OACA investigations that relied solely on unadjusted citation tallies by the explicit inclusion of the RCR as a primary endpoint and the use of robust statistical methods with sensitivity analyses addressing potential confounding.

## Methods

The data comprised all articles that iCite® classified as ‘research articles’ with the Medical Subject Headings (MeSH) of ‘Medical Education’ in the PubMed medical database during the period of 2010−2019 (*N* = 43,275). PubMed is the US National Library of Medicine’s (NLM) large literature database for biomedicine. It serves as a search engine for MEDLINE and other sources and contains over 39 million short references (titles, abstracts, keywords) to biomedical articles. However, PubMed often links to full texts, either on publishers’ websites or to freely accessible versions. Open Access content can be recognised in PubMed by the presence of a link to ‘Free PMC article’. PubMed itself does not host any articles, but makes it much easier to find OA literature. Filters can be used to search specifically for freely available articles, which is important in the medical field so that, for example, doctors can find current studies without expensive journal subscriptions. PubMed has established itself in the medical field as a high-quality but also fast search tool. Studies show that the time factor in particular is decisive in the field of medicine. For example, Daei et al. showed in a systematic review that on average, doctors develop up to 1.5 clinical questions per patient, but leave them unanswered in up to 77% of cases due to a lack of time and access resources [17].

PubMed Central (PMC), on the other hand, is a fully-fledged open access repository for the life sciences, operated by the NLM/NIH. It was established in 2000 as a freely accessible full-text archive for biomedical and life science articles. All content in PMC is available free of charge in full text. PubMed offers direct access to OA articles via the PMCID through its search interface. This study operationalizes OA via the presence of a PMCID, which captures articles from journals with PMC participation agreements, including gold OA journals, as well as author manuscripts deposited under funder mandates. As Piwowar et al. demonstrated, the PMCID serves as a valid identifier for free full-text availability, as it indicates unrestricted access via PubMed Central at the time of query. This conservative definition does not encompass all OA pathways. In particular, gold OA from journals without PMC mirroring, hybrid OA without funder mandates, and bronze OA remain uncaptured. We therefore refer to our operationalized variable as ‘PMCID-indexed OA’ when reporting study-specific results to distinguish it from the broader OA concept.

To test the main hypothesis that OA articles have significantly higher citation metrics than non-OA articles, a retrospective bibliometric cohort study was conducted with a group comparison of ‘PMCID present’ versus ‘PMCID absent’. Methodologically, this follows Davis et al. who even performed a similar comparison in a randomised setting [12]. The cohort size allows robust inferential statistics even with a strongly right-skewed distribution.

Metadata were retrieved in March 2025 via the iCite API (National Institutes of Health) and the PubMed e-utilities interface. As only publicly available data were used, no ethics clearance was required.

The primary endpoints were (1) total citations, (2) citations per year and (3) the Relative Citation Ratio (RCR), as output via the iCite® API. An adjustment for publication year or journal impact factor was deliberately not made. The Relative Citation Ratio (RCR) is a field- and time-normalised citation measure: For each publication, the observed citation rate is benchmarked against an expected rate of articles of the same publication year within its co-citation network [18]. An RCR of 1.0 indicates performance at the median level for NIH-funded papers within the same network. By contrast, an RCR greater than 1.0 denotes above-median influence. The values were retrieved via the iCite® relative_citation_ratio field and analysed in their original form to facilitate direct interpretation.

We selected Yuen-Welch tests over nonparametric alternatives (e.g., Mann-Whitney U) because trimmed means provide a direct, interpretable measure of central tendency that is more informative than rank-based statistics, particularly for right-skewed distributions with heavy tails. The 20% trimming level is conventional in robust statistics, balancing robustness to outliers with statistical efficiency. At 20% trimming, the test remains robust to contamination levels up to approximately 20% while retaining high efficiency under normality.

To address potential confounding, we supplemented primary Yuen-Welch tests with negative binomial regression models including year fixed effects (Model 2) and year plus journal fixed effects (Model 3). The journal impact factor was not included as a covariate in primary analyses because it lies on a plausible causal pathway: prestigious journals may both promote OA options and attract more citations, making adjustment potentially over-controlling. This reasoning is formalised in a directed acyclic graph (Supplement Figure S1). However, Model 3 provides a conservative estimate by absorbing journal-level heterogeneity.

Data transformation was performed in R 4.4.3 [19] using the packages tidyverse [20] for data preparation and statsExpressions [21] for robust tests, MASS for negative binomial regression, and quantreg for quantile regression. As variance heterogeneity and skewness violated the requirements of parametric methods, Yuen-Welch t-tests with 20% trimming were used. The significance level was set at *α* = 0.05, two-sided. Effect sizes were expressed as Algina-Keselman-Penfield robust standardised difference [22]. 95% confidence intervals were determined by bootstrapping.

All data were retrieved from publicly available sources (iCite API, PubMed E-utilities). The R script and detailed API query documentation are provided as Supplementary Materials S6. A frozen data snapshot (parquet format) is available from the corresponding author upon request.

## Results

Of the 56,913 identified medical education publications, 43,275 (76.0%) met the iCite® criteria for research articles. 9,156 (21.2%) of the research articles had a PMCID and were classified as PMCID-indexed OA. The remaining 34,119 articles formed the non-OA reference.

**Figure 1.**
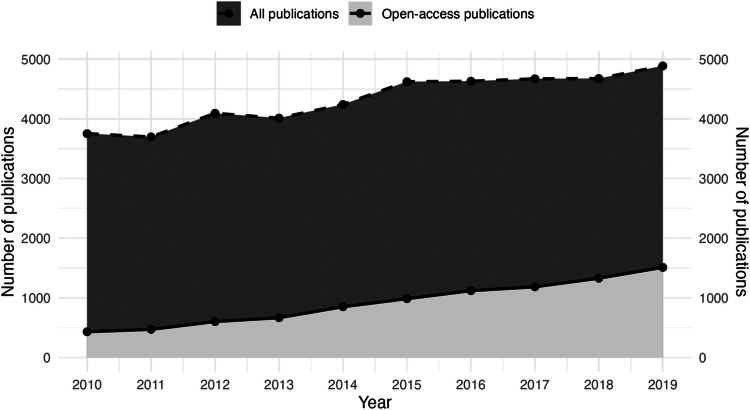
Annual number of PubMed-publications in medical education—Dark-grey area (dashed line) = all research articles (*N* = 43,275) indexed with the MeSH descriptor ‘Medical Education’. Light-grey area (solid line) = open-access subset, defined by the presence of a PubMed Central ID (PMCID). Article-type labels follow the iCite® ‘research article classification.

Figure 1 illustrates the temporal dynamics: While the OA proportion (solid line) increased from 11.5% to 30.9%, the research article curve (dashed line) remained relatively stable from approximately 2015 onward. The proportion of OA articles increased significantly over time (logistic regression: OR = 1.14 per year, 95% CI [1.13, 1.15], *p* < .001). However, the OA × Year interaction on total citations was not significant (*p* = .23), indicating that the citation advantage did not systematically change across the observation period.

The medians for total citations, citations per year and the RCR can be found in Table 1. Table 2 presents trimmed means (20%), untrimmed means, medians, and MAD for each outcome by OA status. All Yuen-Welch test statistics reference the trimmed mean difference. Complete distributional statistics including percentiles are provided in Supplement Table S1.

**Table 1.** Distribution of citation metrics: MAD = Median Absolute Deviation.

Variable	With PMCID (*n* = 9156)	Without PMCID (*n* = 34119)	Total (*n* = 43275)
Median Total Citations (MAD)	9.00 (10.38)	6.00 (7.41)	7.00 (8.90)
Median Citations Per Year (MAD)	1.17 (1.24)	0.67 (0.88)	0.78 (0.99)
Median Relative Citation Ratio (MAD)	0.72 (0.74)	0.42 (0.55)	0.48 (0.61)

**Table 2.** Citation Metrics by Open Access Status^1^.

Metric	Non-OA (*n* = 34,119)	OA (*n* = 9,156)
**Total Citations**		
Trimmed Mean (20%)	7.00	10.31
Untrimmed Mean	13.19	16.22
Median (MAD)	6.00 (7.41)	9.00 (10.38)
**Citations per Year**		
Trimmed Mean (20%)	0.79	1.29
Untrimmed Mean	1.44	1.98
Median (MAD)	0.67 (0.88)	1.17 (1.24)
**Relative Citation Ratio**		
Trimmed Mean (20%)	0.49	0.79
Untrimmed Mean	0.87	1.17
Median (MAD)	0.42 (0.55)	0.72 (0.74)

^1^ MAD = Median Absolute Deviation. Trimmed means use 20% symmetric trimming.

**Figure 2.**
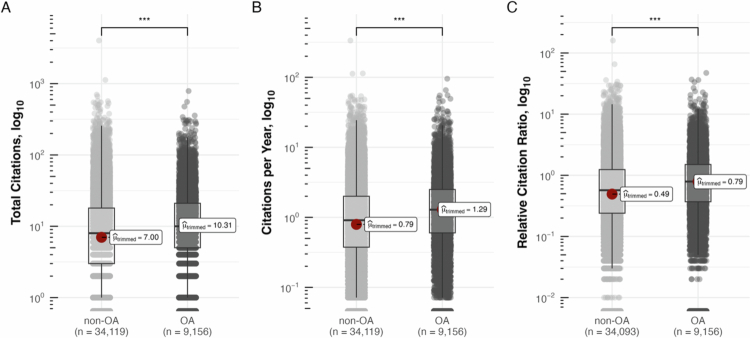
Citation Metrics by open-access status—Boxplot of the citation metrics for PubMed-indexed research articles in Medical Education published between 2010 and 2019. Statistical analyses include all articles, including those with a Relative Citation Ratio (RCR) of 0. Due to the log10-transformation of the y-axis, RCR = 0 values are not visible in the plot, but were retained for all calculations (e.g., trimmed means, significance testing). The central box shows the median and interquartile range; whiskers extend to 1.5 × IQR. Superimposed semi-transparent points represent individual articles. Open-access articles are defined as records carrying a PubMed Central identifier (PMCID); non-open-access articles lack a PMCID. The red dot and dashed line mark the 20% trimmed mean, shown for each group with sample size. Significance symbols: *p ≤ .05, **p ≤ .01, ***p ≤ .001. Total *N* = 43,275 for Panels A–B; *N* = 43,249 for Panel C (26 articles without RCR).

PMCID-indexed articles achieved an (untrimmed) average of 16.22 versus 13.19 citations in the non-OA segment, $t_{\text{Yuen}}\left(8047.97\right)=22.27$, p<.001, with a small to medium robust effect size ${\hat{\delta }}_{R}^{\text{AKP}}=0.27$ (95% CI [0.25, 0.29]; n=43,275; Figure 2A) [23]. For annual citations, the (untrimmed) average gap for OA and non-OA articles was 1.98 versus 1.44, respectively, $t_{\text{Yuen}}\left(7,662.3\right)=28.35$, p<.001, with a small to medium robust effect size ${\hat{\delta }}_{R}^{\text{AKP}}=0.35$ (95% CI [0.33, 0.36]; n=43,275; Figure 2B). The RCR averaged 1.17 for OA versus 0.87 for non-OA articles (untrimmed) and there was a significant difference between the groups, $t_{\text{Yuen}}\left(7,739.61\right)=27.72$, p<.001, with a small to medium robust effect size ${\hat{\delta }}_{R}^{\text{AKP}}=0.34$ (95% CI [0.32, 0.36]; n=43,249; Figure 2C).

Sensitivity analyses using negative binomial regression with year fixed effects confirmed the robustness of these findings (Supplement Table S3). OA status was associated with significantly higher citation counts (IRR = 1.60, 95% CI [1.52, 1.69] for total citations; IRR = 1.53, 95% CI [1.46, 1.61] for citations per year). Models including both year and journal fixed effects yielded similar results (IRR = 1.60, 95% CI [1.51, 1.69]), and restriction to 2016–2019 showed a somewhat stronger association (IRR = 1.67, 95% CI [1.54, 1.80]). Quantile regression indicated that the OA advantage was consistent across the RCR distribution, with estimates ranging from 0.18 at the 25th percentile to 0.44 at the 90th percentile (Supplement Table S4). A sentinel analysis restricted to articles with immediate PMC release (lag ≤ 0 days; *n* = 5,012) confirmed the OA advantage (Supplement Table S5, Panel A), suggesting that immortal time bias does not fully account for the observed effect.

RCR was unavailable for 26 articles (0.06%), all occurring in the non-OA group (χ² = 5.77, *p* = .016). These cases involved articles with very low citation counts (median = 1) published in non-English language journals, where iCite's co-citation network was insufficient for RCR calculation. Because these missing cases would likely have very low RCR values, their exclusion marginally inflates the non-OA group mean, making our estimate of the OA advantage slightly conservative. Given the minimal number affected (0.06%), this differential attrition is unlikely to materially affect our conclusions.

## Discussion

The proportion of PMCID-indexed OA increased steadily between 2010 and 2019 from ≈10% to ≈30%. The analysis shows a consistent, moderate OACA for research papers in the field of medical education. PMCID-indexed articles were cited ≈23% more frequently on average and achieved ≈35% higher RCR values than non-OA publications. This supports the main hypothesis. As the RCR controls for disciplinary citation cultures and publication age by its very nature, this finding provides strong evidence that the open access (OA) advantage in medical education reflects genuine scholarly influence rather than artefacts of topic popularity or historical context.

The observed effect sizes (${\hat{\delta }}_{R}^{\text{AKP}}$  = 0.27–0.35) are conventionally classified as small to medium. However, effect size interpretation should consider the specific context. In bibliometrics, where citation distributions are highly skewed and influenced by numerous factors beyond author control, even modest systematic differences carry practical significance. A 23% increase in citations and 35% higher RCR for PMCID-indexed OA articles, sustained across a decade and 43,000 publications, represents meaningful added visibility for researchers choosing OA. Nonetheless, we emphasise that OA is neither necessary nor sufficient for impact. Excellent non-OA research continues to be widely cited, and OA alone does not guarantee readership.

The proportion of PMCID-indexed OA in the field of medical education is also comparable with the literature. According to a study by Piwowar et al., at least 28% of all scientific literature (≈19 million articles) was already freely accessible 10 years ago [7]. For the most recent publication year examined, 2015, the OA share was already 45% and it grew primarily due to the increases in the gold and hybrid segment [7]. Users of the Unpaywall extension came across a freely available version in 47% of the cases they accessed, which underlines the practical relevance of the OA offer [7] and is also reflected in our results.

Our findings replicate the OACA trend identified by Langham-Putrow et al. with a 23% citation advantage. However, this figure is lower than the ≈31% advantage we calculated from the average relative citation (ARC) counts reported by Piwowar et al. for OA in reference to non-OA, with ARC = 0.90 for paywalled articles and ARC = 1.18 for open-access articles (own calculation: 1.18/0.90 − 1 = 0.311) [5,7]. Not all studies support this finding. A randomised study of articles published by the American Physiological Society showed that OA articles had more reads but did not exhibit OACA [12], whereas Yi et al. confirmed a positive OA effect in the *Postgraduate Medical Journal* [11]. In the systematic literature review conducted by Langham-Putrow et al. [5], the findings were inconsistent, even though the authors focused only on the high-quality studies. Of the three studies with an overall low risk of bias, one confirmed the OACA [24], one denied it [25] and the other found it only in subgroups [26]. The authors also emphasise that studies with a multidisciplinary focus report a partial OACA significantly more often, which indicates context-dependent effects [5]. The lower effect size in our study could be due to the already high baseline citation rate in the field, which Amath et al. describe as characteristic of medical education journals [27].

For context, RCR is specifically designed to enable fair comparisons across fields with different citation practices. Hutchins et al. (2016) demonstrated that RCR distributions are statistically indistinguishable between fields with vastly different raw citation rates, such as basic cell biology and neurological function, confirming successful field normalisation [18]. The median RCR of 0.48 observed in our sample indicates that the typical medical education article is cited approximately half as often as expected based on its co-citation network. This within-field comparison avoids the pitfalls of cross-disciplinary citation comparisons that plagued earlier metrics like the Journal Impact Factor.

Despite growing OA availability, subscription-based publishing persists for several reasons. First, many prestigious society journals (e.g., Academic Medicine, Medical Education) offer hybrid options but remain primarily subscription-based. Stratification by journal type confirmed that the OA advantage was present in both core medical education journals and general medical journals, though somewhat larger in the former (Supplement Table S5, Panel C). Second, APC costs ($2,000–$5,000) create genuine barriers, particularly for unfunded research and authors without institutional support. Third, some fields maintain disciplinary norms that favour publication in established subscription-based society journals (e.g., surgical subspecialty journals), where journal reputation and peer review traditions may outweigh OA considerations in author decision-making. Fourth, author choice typically prioritises journal reputation and peer review quality, with OA status being a secondary consideration, consistent with our causal model where journal prestige influences both OA likelihood and citation outcomes. Finally, the 79% of articles in our sample without PMCIDs may include substantial 'bronze OA' accessible through publisher websites without formal OA licensing—a pathway not captured by our operationalization.

The updated NIH Public Access Policy (effective July 1, 2025) mandates that all peer-reviewed manuscripts from NIH-funded research be publicly available in PubMed Central immediately upon publication, without embargo. This policy shift, occurring after our observation period (2010–2019), may alter the OACA landscape by eliminating the delay between publication and OA availability that currently affects deposit-mandate compliance. Future studies should examine whether immediate-access mandates amplify the citation advantage observed here or whether universal compliance erases the citation differential.

### Limitations

The PMCID-based OA definition used in this study captures only a subset of all open-access variants, primarily green OA deposits in PubMed Central, often mandated by funders such as the NIH. This operationalization captures articles deposited in PMC, which predominantly includes NIH-funded research and articles from journals with PMC participation agreements. It may undercount gold OA from journals without PMC mirroring, hybrid OA without funder mandates, green OA in institutional repositories, and bronze OA. Because PMCID-identified articles are disproportionately linked to well-funded research from high-resource institutions, the observed OACA may partly reflect a funder-prestige confound rather than OA access per se. If gold and hybrid OA articles, which often appear in high-impact journals, were included, the effect size might increase; conversely including bronze OA might attenuate the observed advantage, though the mechanisms remain unclear. Future studies should use Unpaywall or OpenAlex data to differentiate OA pathways. Sensitivity analyses using alternative OA definitions confirmed the robustness of these findings (Supplement Table S2).

A further limitation concerns the timing of OA availability. Articles deposited in PMC after an embargo period may have accumulated citations prior to becoming openly accessible. Without article-level deposit dates, we cannot rule out time-dependent misclassification, whereby the apparent OACA includes citations accrued during the closed-access period. This limitation likely leads to an underestimation of the true OA effect, as citations occurring before deposit would be misclassified as ‘OA citations.’

Due to the retrospective design of this study, it is impossible to determine causality because self-selection could bias the results. Authors may choose open access for manuscripts expected to have a high impact, which could exaggerate the observed association. Additionally, the PMCID is a pragmatic but imperfect OA indicator because ‘bronze OA’—identified by Piwowar et al. as a common variant [7]—is not taken into account. In this case, the publisher allows selected articles to be read free of charge, but they cannot be used for any other purpose. Additionally, high-impact journals may more actively encourage OA publication, attracting authors seeking visibility. This creates potential confounding by journal prestige: articles in prestigious journals may be both more likely to be published OA and more likely to be cited, independent of access model. Another limitation is the potential for early-view bias. OA articles often appear online earlier and can therefore accumulate citations over a longer period. Although an adjustment for publication age was made with citations per year, this method does not capture the publication chronology in detail. Because the OA landscape is evolving rapidly, the strength and direction of the OACA may change over time. Future studies should therefore monitor longitudinal trends and explore possible nonlinear relationships.

### Implications and future directions

Faculty can and should increase the visibility of their educational research by prioritising OA publication funds. However, Abdel-Razig et al. emphasised that fee waivers need to be more transparent and widely available to ensure global equity [2]. Future studies should use prospective designs, differentiate OA types and integrate altmetrics. Brembs et al. proposed decentralised infrastructures to facilitate these analyses. This includes the establishment of a standards body led by the scientific community and the redirection of existing publication funds. Funding organisations should expand their minimum requirements for funded institutions to fund modern infrastructure components that replace or supplement traditional journal functions, thus eliminating misaligned incentives between public funding and scientific interests [28].

Future research should incorporate altmetrics, such as article downloads, readership counts, and social media mentions, to assess whether the OA advantage extends beyond formal citations to broader readership and public engagement. Tracking uptake in evidence syntheses (systematic reviews, meta-analyses) and clinical practice guidelines would provide particularly strong evidence of translational impact, though this requires purpose-built databases not currently available at scale.

## Conclusions

Our findings indicate a moderate citation advantage for PMCID-indexed articles in medical education, consistent with the broader OACA literature. However, this observational association does not establish causation, and authors should weigh multiple factors when choosing publication venues, including journal fit, peer review quality, audience reach, and institutional resources, rather than relying on citation metrics alone. The financial burden of APCs remains a genuine barrier, particularly for early-career researchers and those in resource-limited settings. OA publishing is one tool for maximising research impact, but it is neither the only nor always the best choice for every manuscript.

Is it worthwhile for me as a scientist to publish Open Access? Although our study does not allow a causal conclusion to be drawn, the evidence for a benefit is clear. While the principle of free access to research is broadly supported, the decision to publish OA involves trade-offs between visibility gains, financial costs, and journal fit that each author must weigh individually. Given the countless hours of work that go into every scientific project, access should be as easy as possible for potential readers. Practical experience in well-resourced institutions suggests that once an article is accepted by a prestigious journal, funding is often, though not universally, available to cover the open access fee. However, as Abdel-Razig et al. emphasised, this experience may not generalise to authors from low- and middle-income countries or institutions without dedicated OA funds [2]. Expanded fee waiver programs and transformative agreements are needed to ensure equitable access to OA publishing.

## Supplementary Material

Supplementary_Materials.docx Supplementary_Materials.docx

## Data Availability

Data will be made available upon reasonable request from the corresponding author.
