# Supplementary material for: Is it worth publishing Open Access? – the scientific impact of Open Access publications in the field of medical education
Source: Med Educ Online. 2026 Mar 30;31(1):2652722. doi: 10.1080/10872981.2026.2652722 (PMC13040575; doi:10.1080/10872981.2026.2652722)
Supplement: Supplementary_Materials.docx [file ZMEO_A_2652722_SM1594.docx]

**Supplementary Materials**

*Is it worth publishing Open Access? – The scientific impact of open access publications in the field of medical education*

Hendrik Friederichs

# **Table S1: Extended Descriptive Statistics**

Complete distributional statistics for citation metrics by Open Access status, including percentiles (P25, P75, P90, P95), median absolute deviation (MAD), and trimmed means.

| **Outcome** | **Group** | **n** | **Mean** | **Trimmed Mean** | **Median** | **MAD** | **P25** | **P75** | **P90** | **P95** |
| --- | --- | --- | --- | --- | --- | --- | --- | --- | --- | --- |
| Total Citations | Non-OA | 34,119 | 13.19 | 7.00 | 6 | 7.41 | 1 | 15 | 32 | 48 |
|  | OA | 9,156 | 16.22 | 10.31 | 9 | 10.38 | 4 | 19 | 36 | 53 |
| Citations/Year | Non-OA | 34,119 | 1.44 | 0.79 | 0.67 | 0.88 | 0.17 | 1.70 | 3.50 | 5.17 |
|  | OA | 9,156 | 1.98 | 1.29 | 1.17 | 1.24 | 0.50 | 2.40 | 4.33 | 6.29 |
| RCR | Non-OA | 34,093 | 0.87 | 0.49 | 0.42 | 0.55 | 0.11 | 1.05 | 2.10 | 3.11 |
|  | OA | 9,156 | 1.17 | 0.79 | 0.72 | 0.74 | 0.30 | 1.43 | 2.58 | 3.71 |

*Note. MAD = Median Absolute Deviation. Trimmed Mean = 20% trimmed mean. OA = Open Access (PMCID present). RCR = Relative Citation Ratio.*

# **Table S2: Sensitivity Analyses (Alternative OA Definitions)**

Citation metrics stratified by three different operationalizations of Open Access status. The primary definition (PMCID) was supplemented by alternative definitions to assess robustness of findings.

| **OA Definition** | **n (OA)** | **n (Non-OA)** | **Mean Total Cit. OA** | **Mean Total Cit. Non-OA** | **p-value** |
| --- | --- | --- | --- | --- | --- |
| PMCID (primary) | 9,156 | 34,119 | 16.22 | 13.19 | < .001 |
| Free full text filter | 12,847 | 30,428 | 15.89 | 12.84 | < .001 |
| Combined (PMCID OR publisher OA) | 14,203 | 29,072 | 15.54 | 12.98 | < .001 |

*Note. All comparisons use Yuen-Welch tests with 20% trimmed means. The OA advantage is consistent across all three definitions.*

# **Table S3: Negative Binomial Regression Results**

Negative binomial regression models with journal and year fixed effects. The coefficient for OA status (oa_pmc = TRUE) represents the effect of Open Access on citation counts.

| **Model** | **Estimate** | **SE** | **IRR** | **95% CI** | **p** |
| --- | --- | --- | --- | --- | --- |
| NB: Total Citations | 0.469 | 0.027 | 1.60 | [1.52, 1.69] | < .001 |
| NB: Citations per Year | 0.427 | 0.025 | 1.53 | [1.46, 1.61] | < .001 |
| FE NB: Total Citations | 0.469 | 0.028 | 1.60 | [1.51, 1.69] | < .001 |
| NB: Total Citations (2016-2019) | 0.510 | 0.039 | 1.67 | [1.54, 1.80] | < .001 |

*Note. NB = Negative Binomial. FE = Fixed Effects. IRR = Incidence Rate Ratio (exp(coefficient)). Models 1–2 include year fixed effects only; Model 3 (FE NB) additionally includes journal fixed effects. The restricted model (2016–2019) includes year fixed effects and reduces temporal heterogeneity in OA adoption.*

# **Table S4: Quantile Regression Results**

Quantile regression coefficients for OA status across different quantiles (tau). This analysis captures differences in the upper quantiles where most citation impact occurs.

| **Outcome** | **Quantile** | **Estimate** | **SE** | **95% CI** | **p** |
| --- | --- | --- | --- | --- | --- |
| Total Citations | 0.25 | 2.00 | 0.72 | [0.60, 3.40] | .005 |
|  | 0.50 | 4.00 | 0.43 | [3.16, 4.84] | < .001 |
|  | 0.75 | 5.00 | 0.26 | [4.48, 5.52] | < .001 |
|  | 0.90 | 7.00 | 0.75 | [5.53, 8.47] | < .001 |
| RCR | 0.25 | 0.18 | 0.01 | [0.17, 0.19] | < .001 |
|  | 0.50 | 0.27 | 0.01 | [0.25, 0.29] | < .001 |
|  | 0.75 | 0.34 | 0.02 | [0.30, 0.38] | < .001 |
|  | 0.90 | 0.44 | 0.05 | [0.34, 0.54] | < .001 |

*Note. Quantile regression estimates the OA effect at different points of the citation distribution. The OA advantage increases at higher quantiles, indicating that OA particularly benefits highly-cited articles.*

# **Table S5: Supplementary Analyses**

**Panel A: Sentinel Analysis (Immediate OA Release)**

To address potential immortal time bias, this analysis compares non-OA articles with OA articles that were released in PMC immediately (lag <= 0 days), ensuring that all citations accrued after the article became freely available.

| **Group** | **n** | **Median Total Cit.** | **Median Cit./Year** | **Median RCR** | **p vs. Non-OA** |
| --- | --- | --- | --- | --- | --- |
| Non-OA | 34,119 | 6.00 | 0.67 | 0.42 | — |
| Immediate OA (lag <= 0) | 5,012 | 9.00 | 1.20 | 0.74 | < .001 |

*Note. Immediate OA defined as PMC release date on or before publication date. The OA advantage persisting in this restricted sample suggests that immortal time bias does not fully explain the observed effect.*

**Panel B: PMC Release Lag Distribution**

Distribution of time (in days) between publication and PMC availability for OA articles with available release dates.

| **Statistic** | **First Non-NA Definition** | **Earliest Available Date** |
| --- | --- | --- |
| n with data | 8,847 | 8,847 |
| Median lag (days) | 0 | 0 |
| % immediate (<= 0 days) | 54.7% | 51.5% |
| % within 30 days | 57.4% | 55.2% |
| % within 1 year | 86.9% | 76.8% |

*Note. First Non-NA: Uses DEP > DP > PHST > EDAT priority. Earliest available: Uses minimum of all available dates (conservative). Approximately half of OA articles are available immediately.*

**Panel C: Stratification by Journal Type**

Citation metrics stratified by journal type (Core Medical Education journals vs. General Medical journals).

| **Journal Type** | **OA Status** | **n** | **Mean Total Cit.** | **Median RCR** | **Mean RCR** |
| --- | --- | --- | --- | --- | --- |
| Core MedEd | OA | 4,821 | 18.45 | 0.85 | 1.28 |
|  | Non-OA | 12,547 | 14.21 | 0.48 | 0.92 |
| General Medical | OA | 4,335 | 13.74 | 0.58 | 1.04 |
|  | Non-OA | 21,572 | 12.58 | 0.38 | 0.84 |

*Note. Core MedEd journals: Academic Medicine, Medical Education, Medical Teacher, BMC Medical Education, Advances in Health Sciences Education, Perspectives on Medical Education. The OA advantage is present in both journal categories.*

# **Figure S1: Directed Acyclic Graph (DAG)**

This DAG illustrates our causal assumptions regarding the relationship between Open Access and citations.

DAG Specification (dagitty syntax):

dag { Year -> OA; Year -> Citations; Prestige -> OA; Prestige -> Citations; OA -> JIF; JIF -> Citations; Country -> OA; Country -> Citations }

Key assumptions:

- Journal Impact Factor (JIF) may mediate the OA-Citation relationship

- Publication year affects both OA probability (increasing trend) and citation accumulation time

- Author country/institution affects both OA probability (funding) and citations (prestige networks)

- Adjusting for JIF may induce overadjustment if JIF lies on the causal pathway

Minimal sufficient adjustment set: Year, Country (if mediator assumption for JIF is accepted)

# **S6: Data Documentation**

**Data Pipeline Script**

The data pipeline script is provided as a separate file (supplement_S6_data_pipeline.R). The complete analysis script including all figures and statistical models is available upon reasonable request from the corresponding author.

Software environment: R version 4.4.3, with packages tidyverse, statsExpressions, MASS, quantreg, and easystats.

**Data Dictionary**

The analysis dataset contains the following key variables:

| **Variable** | **Type** | **Description** |
| --- | --- | --- |
| pmid | character | PubMed ID (unique identifier) |
| pmcid | character | PubMed Central ID (if available) |
| year | integer | Publication year (2010-2019) |
| journal | character | Journal title |
| total_citations | numeric | Total citation count from iCite |
| citations_per_year | numeric | Citations per year since publication |
| rcr | numeric | Relative Citation Ratio (field-normalized) |
| oa_pmc | logical | TRUE if PMCID present (primary OA definition) |
| oa_altA | logical | Alternative OA: PubMed Free full text filter |
| oa_altB | logical | Alternative OA: PMCID OR publisher OA |

**Data Availability**

The analysis dataset will be made available upon reasonable request from the corresponding author. The dataset can also be reconstructed using the provided query strings and the iCite/PubMed APIs.
